# Role of mTORC1 Controlling Proteostasis after Brain Ischemia

**DOI:** 10.3389/fnins.2018.00060

**Published:** 2018-02-15

**Authors:** Maria J. Perez-Alvarez, Mario Villa Gonzalez, Irene Benito-Cuesta, Francisco G. Wandosell

**Affiliations:** ^1^Centro de Biología Molecular Severo Ochoa, CSIC-UAM, Madrid, Spain; ^2^Departamento de Biología (Fisiología Animal), Facultad de Ciencias, Universidad Autónoma de Madrid, Madrid, Spain; ^3^Centro de Investigación Biomédica en Red Sobre Enfermedades Neurodegenerativas, Madrid, Spain

**Keywords:** signaling, PI3K-Akt, autophagy, stroke, neuroinflammation, neuronal stress response, glia stress response, MCAO

## Abstract

Intense efforts are being undertaken to understand the pathophysiological mechanisms triggered after brain ischemia and to develop effective pharmacological treatments. However, the underlying molecular mechanisms are complex and not completely understood. One of the main problems is the fact that the ischemic damage is time-dependent and ranges from negligible to massive, involving different cell types such as neurons, astrocytes, microglia, endothelial cells, and some blood-derived cells (neutrophils, lymphocytes, etc.). Thus, approaching such a complicated cellular response generates a more complex combination of molecular mechanisms, in which cell death, cellular damage, stress and repair are intermixed. For this reason, animal and cellular model systems are needed in order to dissect and clarify which molecular mechanisms have to be promoted and/or blocked. Brain ischemia may be analyzed from two different perspectives: that of oxygen deprivation (hypoxic damage *per se*) and that of deprivation of glucose/serum factors. For investigations of ischemic stroke, middle cerebral artery occlusion (MCAO) is the preferred *in vivo* model, and uses two different approaches: transient (tMCAO), where reperfusion is permitted; or permanent (pMCAO). As a complement to this model, many laboratories expose different primary cortical neuron or neuronal cell lines to oxygen-glucose deprivation (OGD). This *ex vivo* model permits the analysis of the impact of hypoxic damage and the specific response of different cell types implicated *in vivo*, such as neurons, glia or endothelial cells. Using *in vivo* and neuronal OGD models, it was recently established that mTORC1 (mammalian Target of Rapamycin Complex-1), a protein complex downstream of PI3K-Akt pathway, is one of the players deregulated after ischemia and OGD. In addition, neuroprotective intervention either by estradiol or by specific AT2R agonists shows an important regulatory role for the mTORC1 activity, for instance regulating vascular endothelial growth factor (VEGF) levels. This evidence highlights the importance of understanding the role of mTORC1 in neuronal death/survival processes, as it could be a potential therapeutic target. This review summarizes the state-of-the-art of the complex kinase mTORC1 focusing in upstream and downstream pathways, their role in central nervous system and their relationship with autophagy, apoptosis and neuroprotection/neurodegeneration after ischemia/hypoxia.

## mTORC: role in adult central nervous system (CNS)

As mentioned previously, in this review we try to summarize the state-of-the-art of the complex kinase mTORC1 in the brain. We will present some general structural features, and some of elements defined, so far, as belonging to this complex.

We will recapitulate some of the most representative upstream and downstream pathways and their role in central nervous system. We will revise their connexion of mTORC1 with autophagy, apoptosis and neuroprotection/neurodegeneration, mostly focused on ischemia/hypoxia. We are aware that we can only mention some of the published works, due to the lack of space.

### mTORC1/2 structure and function

Mammalian target of rapamycin (mTOR) is a protein with serine-threonine kinase activity that is part of two different multiprotein complexes which differ in some of their components and their upstream and downstream signaling (Takei and Nawa, 2014; Bockaert and Marin, 2015; Switon et al., 2017). This kinase regulates essential aspects of cell function including growth, differentiation, survival and energy homeostasis by harmonizing anabolic and catabolic processes, with the aim of maintaining cellular physiology.

Structurally, mTOR is a multi-domain protein comprised of a C-terminal FAT domain (FATC), a C-terminal kinase domain (KD), a rapamycin binding domain (FRB), a transactivation/transformation-associated domain (FAT) and an N-terminal domain which provides a site of regulatory protein interaction (HEAT repeats) (Hwang and Kim, 2011; Yang et al., 2013; Switon et al., 2017). This HEAT repeats domain allows interaction between mTOR and regulatory-associated proteins, forming two complexes known as mTORC1 and mTORC2 which function as homodimers (Yip et al., 2010; Aylett et al., 2016), and include common and specific proteins. The common regulatory components of mTORC1/2 are mammalian lethal with SEC13 protein 8 (mLST8), Tel two-interacting protein 1 (Tti1), telomere maintenance 2 (Tel2), DEP domain-containing mTOR-interacting protein (DEPTOR) and the kinase mTOR (Yip et al., 2010; Switon et al., 2017). There are two regulatory-associated proteins specific to mTORC1 [regulatory associated protein of Tor (Raptor) and proline-rich AKT1 substrate 40 kDa (PRAS40)] whereas the characteristic proteins of mTORC2 are rapamycin-insensitive companion of mTOR (Rictor), mammalian stress-activated protein kinase-interacting protein 1 (mSin1), and protein observed with Rictor (PROTOR).

Consequently, mTORC1 and mTORC2 differ not only in the composition of their specific regulatory proteins but also in their rapamycin sensitivity, mediated by FK506-binding protein (FKPB12). In fact, FKPB12 is another mTOR-interacting protein that, in presence of rapamycin, blocks the catalytic domain of mTORC1, reducing its kinase activity. In contrast, mTORC2 does not interact with FKBP12-rapamycin, and is thus rapamycin-insensitive. Nevertheless, sustained treatment with rapamycin inhibits mTORC2 activity, probably by sequestration of mTOR protein or uncoupling of mTORC2 components such as SIN1 (Sarbassov et al., 2006; Chen and Sarbassov, 2011; Switon et al., 2017).

**Figure 1:**
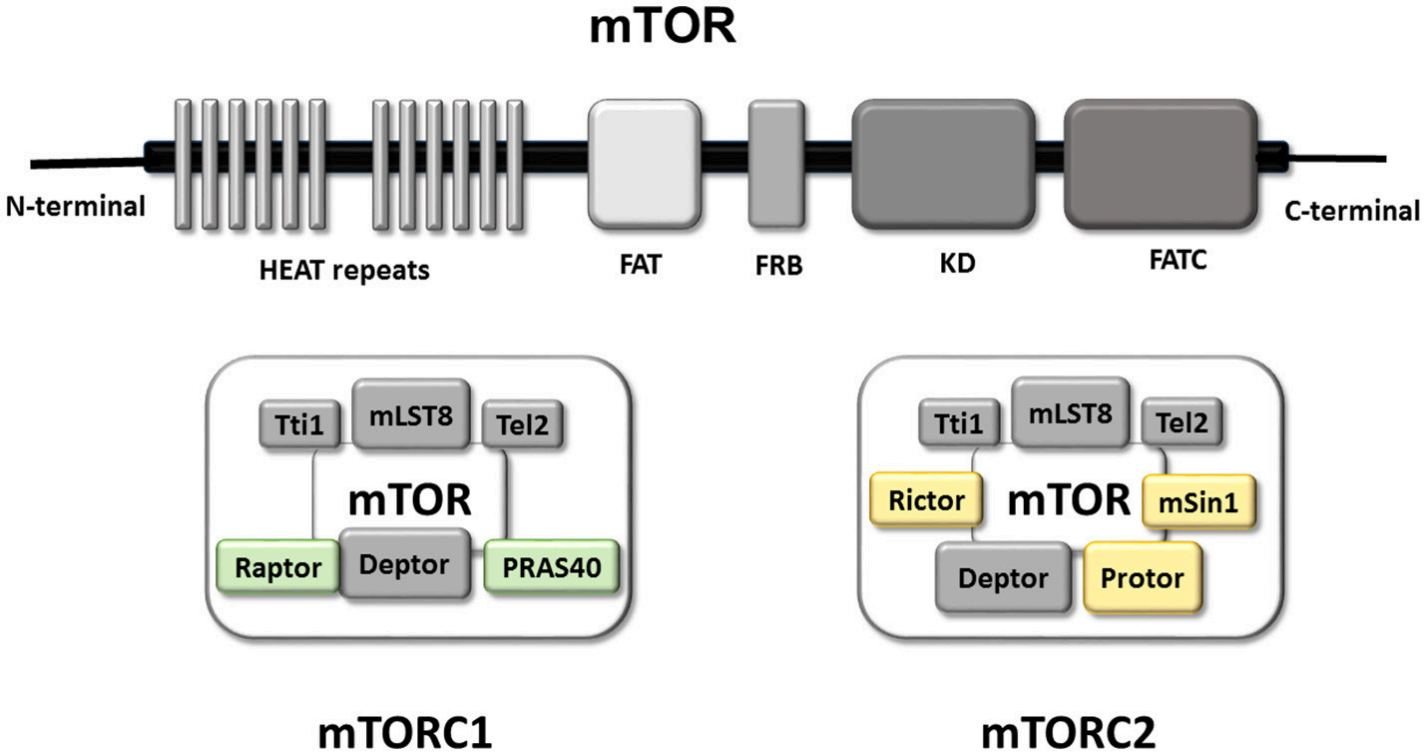
mTOR Functional domains and composition of mTORC1 and mTORC2. Graphical representation of the main functional domains of mTOR protein. mTOR contains at C-terminal a FAT domain (FATC), the kinase domain (KD), a rapamycin binding domain (FRB) and a transactivation domain-associated protein domain (FAT). The N-terminal domain is composed by a HEAT repeats site with a key role in the interaction with regulatory proteins permitting the formation of mTOR complex (mTORC1 right and mTORC2 left). Representation of the mTORC1 and mTORC2 complex Both complex are formed, so far, by five common proteins and two/three specific proteins. The common components of both complexes are in gray (Tti1; mLST8; Tel2; Deptor in gray). Whereas Raptor and PRAS40 are present in mTORC1 (green), and Rictor, Protor and mSin1 are part of mTORC2 (yellow).

The role of each regulatory protein in the activity of mTORC1/2 is not completely understood. Studies using mLST8-deficient mice have revealed that mLST8 is necessary to maintain Rictor-mTOR but not Raptor-mTOR interactions, and consequently perturbs mTORC2 but not mTORC1 activity (Guertin et al., 2006). Tti1 and Tel2 are important components for assembly and maintenance of mTORC1/2 activity, and are also critical for mTOR stability (Kaizuka et al., 2010). As DEPTOR is an endogenous inhibitor of mTOR, its abundance modulates activity of mTORC1/2, and it is found to be overexpressed in some cancer types (Peterson et al., 2009; Liu et al., 2010; Catena and Fanciulli, 2017). PRAS40 is a negative regulator of mTOR, preventing binding of mTORC1/2 to its substrate (Wang et al., 2007). Raptor is important for mTORC1 assembly, regulates mTORC1 activity and localization, and plays a key role in the recruitment of mTORC1 substrates, the best-characterized of which are translational regulators (Hara et al., 2002; Yonezawa et al., 2004; Aylett et al., 2016). Whereas Rictor and mSin1 stabilize each other and constitute the structural basis of mTORC2, Rictor also gives mTORC2 substrate specificity (Dos et al., 2004). PROTOR doesn't affect mTORC2 assembly, but plays a role modulating mTORC2 kinase efficiency (Pearce et al., 2007, 2011; see Figure 1).

In addition to these regulatory proteins, mTORC1 activity is regulated by phosphorylation of specific C-terminal Ser/Thr residues, performed by different kinases such as: Akt, p70 ribosomal S6 kinase (p70S6K), AMP-activated protein kinase (AMPK), and also possesses an autophosphorylation site (Chong et al., 2013). Therefore, the activity of mTORC1 is highly regulated indicating the importance of maintaining mTORC1 activity levels within a physiological range. Indeed, mTORC1 is considered to be a nutrient-sensitive complex that regulates cellular growth, protein synthesis and autophagy, whereas mTORC2 has been defined as a regulatory kinase of Akt/PKB and thus a component of growth factor signaling, although mTORC2 has not been characterized as fully as mTORC1.

From a functional viewpoint, mTORC1 is localized at a strategic position in signaling pathways. On one hand, its activity is modulated upstream by several pivotal intra- and extracellular metabolic and energetic factors including growth factors, amino acids, cell energetic status and oxygen availability, through different pathways (Weichhart, 2012; Dibble and Cantley, 2015; Chen et al., 2017; Switon et al., 2017). Thus, mTORC1 can be considered to be a molecular sensor for essential information related to nutrient availability, growth factors and energetic balance. Additionally, mTORC1 modulates important cellular processes such as translation, transcription, lipid synthesis, autophagy and the cell cycle (Pla et al., 2016; Ben-Sahra and Manning, 2017; Hong et al., 2017; Liu et al., 2017).

It is currently accepted that activation of the kinase activity of mTOR increases the rate of protein synthesis by both activating the processes of translation initiation and elongation in cells. In fact, two of the best know targets of mTORC1, p70S6K and 4E-binding proteins (4EBPs), have important roles in two phases of the translation machinery: initiation and elongation (Chong et al., 2013).

### mTORC1/2 and CNS

Expression of mTOR in the adult brain is high, predominantly in neurons but also in glial cells (Perluigi et al., 2015). However, most of the knowledge regarding the neurologic role of mTORC1 comes from studies using neurons, and only limited information related to glial cells is available. In neurons, mTORC1 activity is modulated by neuron-specific molecules including neurotransmitters (e.g., glutamate, dopamine, serotonin, GABA, cannabinoids), and neurotrophic factors [e.g., brain-derived neurotrophic factor (BDNF), insulin-like growth factor 1 (IGF-1)] (Bermudez-Silva et al., 2016; Chen et al., 2017). Some important processes related to development and maturation of the CNS are controlled by mTORC1 including neurogenesis, axonogenesis, axon guidance, dendritogenesis, and growth of dendritic spines (Jaworski and Sheng, 2006). In addition, mTORC1 plays a key role in adult brain physiology and pathology, influencing higher brain functions such as learning, memory (Jaworski and Sheng, 2006), feeding behavior (review in: Cota et al., 2006; Woods et al., 2008), synaptic plasticity (Bockaert and Marin, 2015), cognition (Burket et al., 2014), circadian rhythm (Jouffe et al., 2013) and sensorial perception by modulation of sensitivity to peripheral sensory afferents (Obara and Hunt, 2014).

Similarly, mTORC1 plays a role in gliogenesis during brain development (Cloëtta et al., 2013), and has also been implicated in differentiation and maturation of several glial cell types in the adult brain, especially in oligodendrocytes and microglia (Tyler et al., 2009). Recent data emphasizes the role of mTORC1 in the control of lipid biosynthesis in oligodendrocytes and Schwann cells, through sterol regulatory element-binding proteins (SREBPs). Consequently, its contribution to correct peripheral nerve function is related to velocity of action potential conduction and precise brain myelination (Norrmén et al., 2014; Zou et al., 2014). The role of mTORC1 in the balance of M1/M2 microglia phenotype has been reported (Chen et al., 2016), and reduction of mTORC1 activity decreased pro-inflammatory cytokines and chemokine synthesis, reducing the M1 microglia phenotype (Li et al., 2016). Also, mTORC1 has been found to be important for survival and size preservation of astrocytes (Pastor et al., 2009).

Taking into account the broad cerebral functions regulated by mTORC1 and its central position in cellular energetic/metabolic homeostasis, dysregulation of its activity (increasing or decreasing) would be detrimental for normal brain physiology. Recent evidence supports that modification of mTORC1 activity can trigger diseases of the nervous system, since disruption in mTORC1 signaling affects multiple pathways including energy production, mitochondrial function, cell growth, glucose/lipid metabolism and autophagy.

Mutations in the genes encoding different molecular components of mTORC1 or its related signaling pathways result in diseases characterized by severe neurological symptoms including epilepsy, tumor growth, autism and cognitive disability. Use of transgenic animals has enabled correlation of mTORC1 dysregulation with neurological indicators (for a revision see Switon et al., 2017). Some human genetic disorders have been associated with disturbed mTORC1 activity, including Tuberous Sclerosis complex, currently considered mTORopathy. It is a multi-system autosomal-dominant disorder characterized by benign tumors in some systemic organs, cortical and cerebellar tuber, subependymal nodules, retinal hamartomas, and hypomyelination (Carson et al., 2015; Mühlebner et al., 2016; Hodgson et al., 2017). Clinical manifestations include epilepsy, mental disability and autism (Zaroff et al., 2004; Mühlebner et al., 2016). This disorder is caused by mutations in TSC1 and TSC2 that encode Tuberous Sclerosis Complexes (Tsc1 and Tsc2) that belong to the canonical pathway of mTORC1 activation by trophic factors (see below), whereas these Tsc1/2 mutations induce mTORC1 hyperactivation.

Neurofibromatosis is caused by mutations in the gene that encodes neurofibromin, a modulator of mTORC1 activity (Giovannini et al., 2014). It is characterized by the appearance of tumors in the central and peripheral nervous system and anomalies in other tissues (skin, kidney, and bone). Clinical symptoms consist of learning disabilities, epilepsy and anxiety among other. Analysis of human samples has demonstrated that mTORC1 is over-activated, though administration of rapamycin in *in vitro* and *in vivo* models reduced the severity of tumors without toxicity (Giovannini et al., 2014).

In the adult brain, maintaining a precise balance of protein translation and degradation (proteostasis) is fundamental to avoid accumulation of toxic protein aggregates and oxidized proteins that might trigger brain degeneration, such as in Alzheimer's disease (AD), Parkinson's (PD), or Huntington's (Ht). Indeed, mTORC1 is an important player in proteostasis, through its capacity to control translation and autophagy.

In some neurodegenerative disorders such as AD or PD, characterized by the anomalous accumulation of aggregated misfolded proteins, data strongly suggests an anomalous level of mTOR-dependent autophagy. In fact, the reduction of autophagy with aging is a key mechanism that may contribute to the accumulation of protein aggregates in neurons (for a review see: Dazert and Hall, 2011; Perluigi et al., 2015). Following this hypothesis, it has been demonstrated in some animal models of AD or Ht that administration of rapamycin promotes elimination of toxic, misfolded proteins through induction of autophagy, reducing disease severity (Spilman et al., 2010; Kiriyama and Nochi, 2015; Benito-Cuesta et al., 2017).

Cerebral ischemia is an unexpected injury that may trigger, among other things, neuronal and glial cell death, and later neuronal degeneration (explained in detail below).

Our “working hypothesis” is that mTORC1 may play an important role in limiting ischemic damage. Several evidences sustain this assumption: (i) mTORC1 has the capacity to control anabolic/catabolic cell processes; (ii) mTORC1 potentially modulate neuronal apoptosis and autophagy; and (iii) considering the role of mTORC1 promoting neurogenesis and improving angiogenesis. Consequently, we propose mTORC1 as a potential therapeutic target for ischemic stroke.

In this review, we discuss the role of mTORC1 with an emphasis on the current knowledge about its impact in brain ischemia, analyzing recent evidence from both *in vivo* and *in vitro* models of cerebral ischemia.

## Signaling pathways upstream of mTORC1

The level of activation of mTORC1 depends on integration of extracellular signals through different pathways, permitting the combination of multiple events to generate a suitable response in order to maintain cellular homeostasis. In this section we explain the key players in signaling upstream of mTORC1.

### The canonical pathway: PI3K/Akt/mTOR

As a general rule, growth factors bind to two types of receptors, Tyrosine kinase receptors (RTKs) or G-protein-coupled receptors (GPCRs). Downstream of both, activation of class I PI3-kinase is an essential step for initiating the signaling cascades. The activation of this lipid kinase generate a plethora of responses such as PDK1/Akt pathway, among others.

It is well known that complete activation of Akt requires the action of two different kinases (pyruvate dehydrogenase kinase 1, PDK1 and mTORC2) at two respective amino acid residues: Thr308 and Ser473 (Zhao et al., 2006; Dibble and Cantley, 2015). Downstream, Tsc has been identified as the connection between Akt and mTORC1, and is formed by three proteins: Tsc1, Tsc2, and TBC1D7 (Dibble and Cantley, 2015). Tsc1 (also called Hamartin) is a stabilizing factor of Tsc2. While Tsc2 (named Tuberin) is a guanosine triphosphatase (GTPase)-activating protein (GAP) toward Ras homolog enriched in brain protein (Rheb).

Akt phosphorylates Tsc2 and induces its dissociation from Tsc1 (Dibble and Cantley, 2015). Activation of Tsc1/2 inactivates the GTPase Rheb, which functions as a molecular switch by alternating between GTP and GDP-bound forms (Parmar and Tamanoi, 2010). GTP-Rheb directly activates mTORC1 (Malik et al., 2013), but Tsc1/2 action converts GTP-Rheb into GDP-Rheb, inactivating mTORC1. The loss of Tsc1/2 function is a determinant of mTORC1 over-activation that results in brain disease independent of growth factors (Frindlay et al., 2005; Hwang and Kim, 2011). Tsc2 activity can also be regulated by kinases such as mitogen-activated protein kinase (MAPK) and AMP-activated protein kinase (AMPK). In fact, AMPK activated by low cellular energy status (increased ratio of AMP/ATP) can phosphorylate Tsc2 at different residues than Akt; whereas phosphorylation of Thr1462 or Ser664 by Akt or MAPK, respectively, inhibits Tsc and activates mTORC1 (Ma et al., 2007), phosphorylation of Ser1345 or Ser1337/Ser1341 by AMPK or GSK3β, respectively, increases Tsc1/2 activity and consequently inhibits mTORC1 (Inoki et al., 2006; Takei and Nawa, 2014).

Thus, the AMPK-dependent pathway counterbalances the effects of trophic factors on mTORC1 activity as long as cellular energy is below an “optimal level” for survival.

### Hormones, growth factors and neurotransmitters

**Figure 2:**
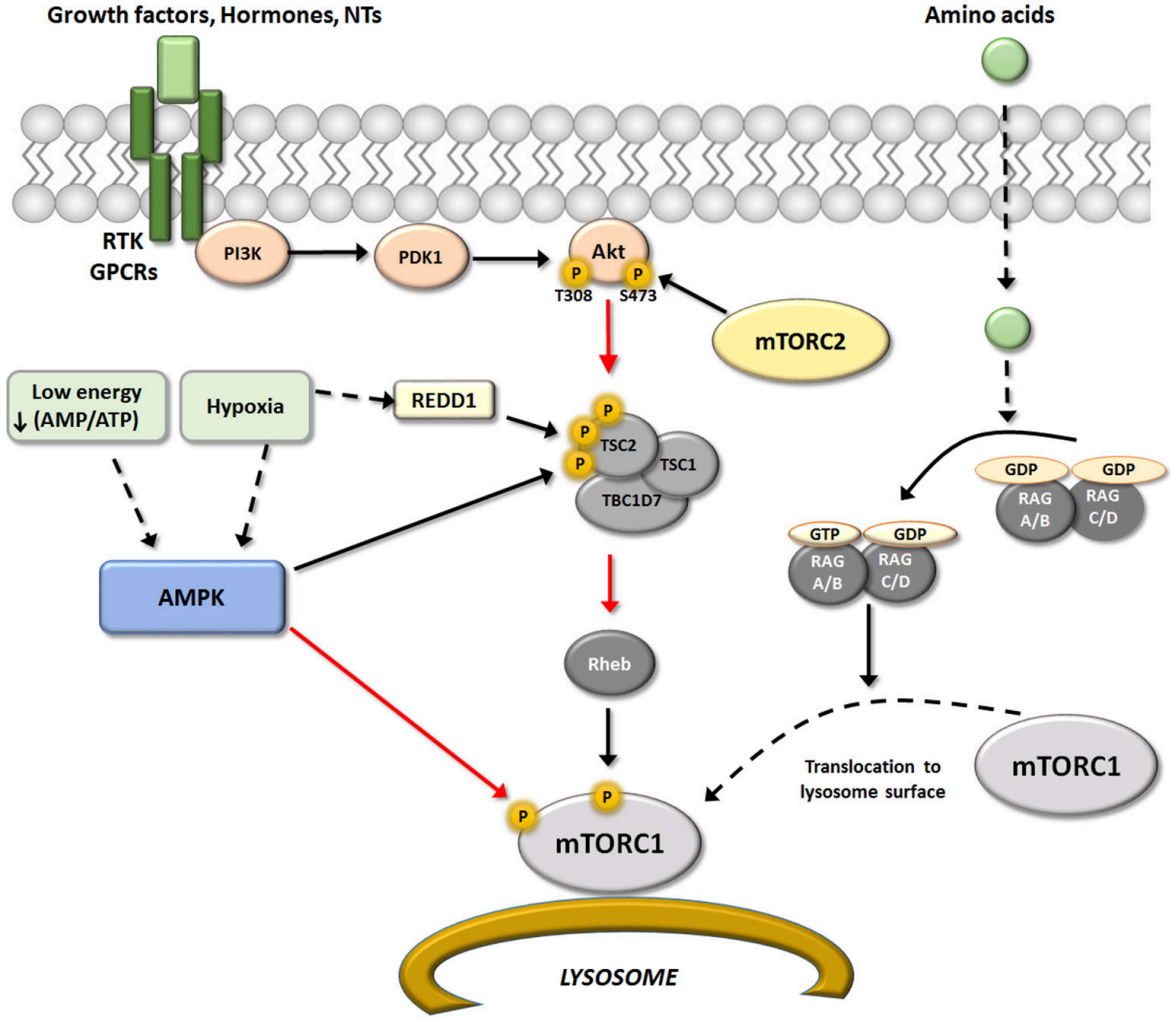
Upstream regulators of mTORC1. Schematic representation of the different upstream pathways of mTORC1. The canonical pathway regulated mTORC1 activity through RTK/GPCRs receptors via modulation of PI3K/PDK1/Akt/TSC/Reb. This pathway may be positively regulated by growth factors, hormones and some neurotransmitters, and negatively regulated by AMPK. AMPK is activated in situations of low energy and/or hypoxia resulting in an inhibition of mTORC1 activity that may occurs at two levels. One by direct Raptor phosphorylation, that result in an inhibition of mTOR activity (red arrow); and second modifying TSC/REDD1 complex by phosphorylation. In addition, amino acids levels could modulated mTORC1 activity directly at the lysosome surface (an important place for activation of mTORC1) by modulation of heterodimers Rag A-D. Black arrows represent activation and red arrows represent inhibition.

Hormones and growth factors like insulin, IGF-1, epidermal growth factor (EGF), neurotrophins [NGF, BDNF and neurotrophin 3/4 (NT-3; NT-4)], or some neurotransmitters, induce activation of downstream effectors of PI3K, Akt and subsequently mTORC1, through binding of specific membrane receptors (Switon et al., 2017; see scheme in Figure 2). Whereas, insulin/IGF-1 and neurotrophins are the best characterized growth factors that activate mTORC1 complexes by the PI3K/Akt pathway (Takei and Nawa, 2014; Chen et al., 2017).

In the nervous system, axonal guidance molecules such as Reelin, Semaphorin, Ephrins, and Netrin-1 have been shown to activate mTORC1 through PI3K/Akt, and also some neuronal receptors like Glu receptors, cannabinoid receptors (CBRs), angiotensin receptors (AT2R), and μ-opioids, among others (Polakiewicz et al., 1998; Page et al., 2006; Puighermanal et al., 2009; Mateos et al., 2016). In some cases, this activation mediates important brain processes such as synaptic plasticity.

### Amino acids

Besides growth factors, some amino acids (not only glutamate and aspartate as neurotransmitters) have pivotal roles in the regulation of mTORC1 activity. Experimental evidence suggests that amino acids, particularly leucine, serve as extracellular stimuli that modulate mTORC1. The connection between mTORC1 and amino acid levels is the Rag family of small G-proteins (Rag A-D) (Kim et al., 2008; Sancak et al., 2008). RagA-D proteins are heterodimers that locate to the lysosome surface, an important site of mTORC1 activation. The active conformations of this GTP/GDP binding protein class are GTP-RagA/B and GDP-RagC/D (Dibble and Manning, 2013; see scheme in Figure 2).

A reduction of amino acid availability induces the GDP-RagA/B form, which triggers detachment of Rag from the lysosomal surface and consequently inactivates mTORC1. In contrast, when amino acid supply is sufficient, Rag binds GTP and undergoes conformational change, directly binding to Raptor and recruiting mTORC1 to the lysosomal membrane (Jewell et al., 2013). This situation allows close proximity between mTORC1 and Rheb on the lysosome surface, which can activate mTORC1. However, the mechanism by which other amino acids may modulate mTORC1 activity is not completely understood (Saxton and Sabatini, 2017).

### Nutrients, energy status and stressful conditions

Energetic status, nutrient availability, and related stress conditions such as glucose deprivation, hypoxia, and DNA damage can modify mTORC1 activity (Shimobayashi and Hall, 2014). In the brain, both glia and neurons use glucose as a primary energy source, and are thus highly sensitive to fluctuations in blood glucose levels (Poels et al., 2009). Hypoglycemia may diminish ATP levels in glia/neurons and increase reactive oxygen species (ROS), triggering AMPK and promoting metabolic reprogramming. As discussed before, AMPK is sensitive to cellular energy status, sensing the AMP/ATP ratio and directing mTORC1 to modulate Tsc1/2 as a compensatory mechanism to preserve cellular energy pools. Activation of AMPK inhibits mTORC1 activity by two pathways, each of which involves phosphorylation of different substrates: Tsc2 and Raptor (Agarwal et al., 2015).

Similarly, a reduction in oxygen availability (hypoxia) inhibits mTORC1 through multiple pathways, especially when hypoxic conditions are sustained over time. Hypoxic activation of Tsc complex occurs by two pathways, one being AMPK-dependent, and the other acting via REDD 1. The increment in REDD1 after hypoxia is enough to liberate Tsc2 from chaperone 14-3-3 and permit its association with Tsc1. Hypoxic conditions may also reduce mTORC1 activity through other proteins that interfere with Rheb/mTORC interaction, including promyelocytic leukemia tumor suppressor (PML) and hypoxia-inducible proapoptotic protein BNIP3 (Brugarolas et al., 2004; Wouters and Koritzinsky, 2008; see scheme in Figure 2).

In summary, the precise impact of amino acids, glucose, growth factors, and neurotransmitters on mTORC1 activity in both physiological and pathological brain situations is not fully understand. This is partly because there isn't much information about the effects of selective elimination of each factor; thus, more work must be performed in cellular and animal models to clarify this issue.

## Signaling pathways downstream of mTOR

The ability of mTORC1 sensing energy and nutrient levels makes it a central node in regulation of metabolism. In nutrient-rich conditions, mTORC1 kinase activity promotes anabolic pathways (translation, transcription and lipid synthesis) and downregulates cellular catabolic processes (protein degradation).

### Anabolic metabolism regulated by mTORC1

The best-identified anabolic process mediated by mTORC1 is protein synthesis, which consumes large amounts of energy and consequently is highly regulated. Activation of mTORC1 (when cellular nutrient and energy levels are optimal) favors protein synthesis. As mentioned previously, p70S6K and 4EBPs are key targets of mTORC1, and are phosphorylated at various sites (p70S6K1 at Thr389; 4EBP at several sites), favoring 5′ cap-dependent mRNA translation. In addition, mTORC1 upregulates several steps of ribosome biogenesis including transcription of ribosomal RNA and ribosomal protein synthesis (Iadevaia et al., 2014). Correct generation of ribosomes is necessary for cellular protein synthesis in general, which is critical for cell survival.

Recently, several studies have focused on the role of mTORC1 in mRNAs translation location-dependent. As neurons are long cells, some proteins must be produced locally on demand at synapses, sometimes far away from cellular soma. This is an important process that contributes to synaptic plasticity (Jaworski and Sheng, 2006; Urbanska et al., 2012). Transcripts activated by mTORC1 in response to BDNF have been described in synaptoneurosomes prepared from cortical neurons, some encoding proteins with important roles in dendritic spines (Panja and Bramham, 2014).

Stress factors such as glucose reduction and insufficient amino acids induce inhibition of mTORC1, promoting diminished phosphorylation of 4EBP1. In such conditions, 4EBP1 associates with the cap-binding eukaryotic translation initiation factor 4E (EIF4E), which prevents EIF4E/EIF4G interaction and therefore prevents cap-dependent translation (Wouters and Koritzinsky, 2008). In conclusion, mTORC1 has a pivotal role modulating 5′ cap-dependent translation, but also mediates translation independently of this canonical pathway. A role has been described for mTORC1 in cap-independent translational control of some critical factors in the brain such HIF-1α, VEGF, and IGF2 (Dai et al., 2011), a mechanism that would maintain expression of important factors during stressful situations. This cap-independent translation happens despite the absence of mTORC1 phosphorylation of signal transducer and activator of transcription 3 (STAT3), which diminishes the transcription of some proteins that use the cap-independent translation mechanism (Dodd et al., 2015).

In addition to its effect on protein synthesis, mTORC1 induces *de novo* nucleotide synthesis through different mechanisms (for a review, see: Ben-Sahra and Manning, 2017). Nucleotide synthesis is not only necessary for ribosome biogenesis but also for cell growth; several reports have indicated that the impact of mTORC1 on nucleotide synthesis is mediated by p70S6K1-dependent phosphorylation (Ben-Sahra et al., 2016). mTORC1 also activates SREBP, a transcription factor that induces expression of genes involved in fatty acid and cholesterol biosynthesis. This is essential to supply lipids for membrane elongation in growing cells, not only in neurons but also in glial cells and oligodendrocytes for myelination of the nervous system (Norrmén et al., 2014).

**Figure 3:**
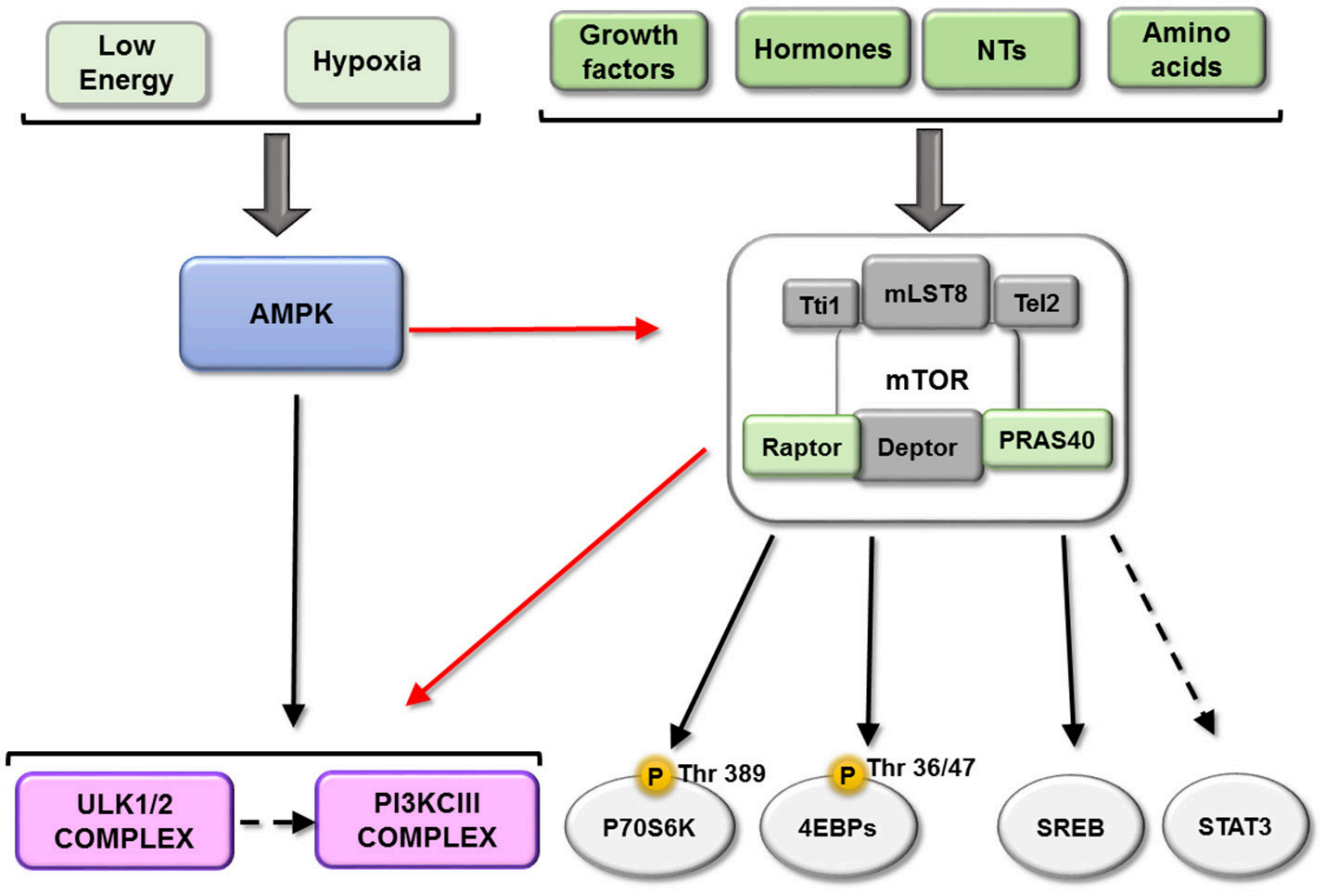
Downstream of mTORC1. mTORC1 play a key role integrating information about the availability of some important metabolic factors. This figure represents the main mTORC1substrates described until now: P70S6K, EBPs, ULK complex and PI3KIII and their relationship. Black arrows represent activation and red arrows represent a inhibition.

All of this evidence shows that mTORC1 tightly coordinates the biosynthesis of the three key macromolecular components of the cell through parallel pathways (see scheme in Figure 3).

### Catabolic metabolism regulated by mTORC1

Autophagy is the catabolic mechanism by which dysfunctional or unnecessary cellular components are degraded through the action of lysosomes (Mizushima and Komatsu, 2011; Boya et al., 2013), and three major subtypes have been described: chaperone-mediated autophagy, microautophagy and macroautophagy. Macroautophagy consists of the formation of an isolated membrane to sequester a portion of cytoplasm within a double membrane vesicle or autophagosome, which subsequently fuses with the lysosome to degrade its content. Apart from the upregulation of macromolecule synthesis, mTORC1 is also able to suppress catabolism, mostly via macroautophagy (hereafter referred to simply as autophagy).

**Figure 4:**
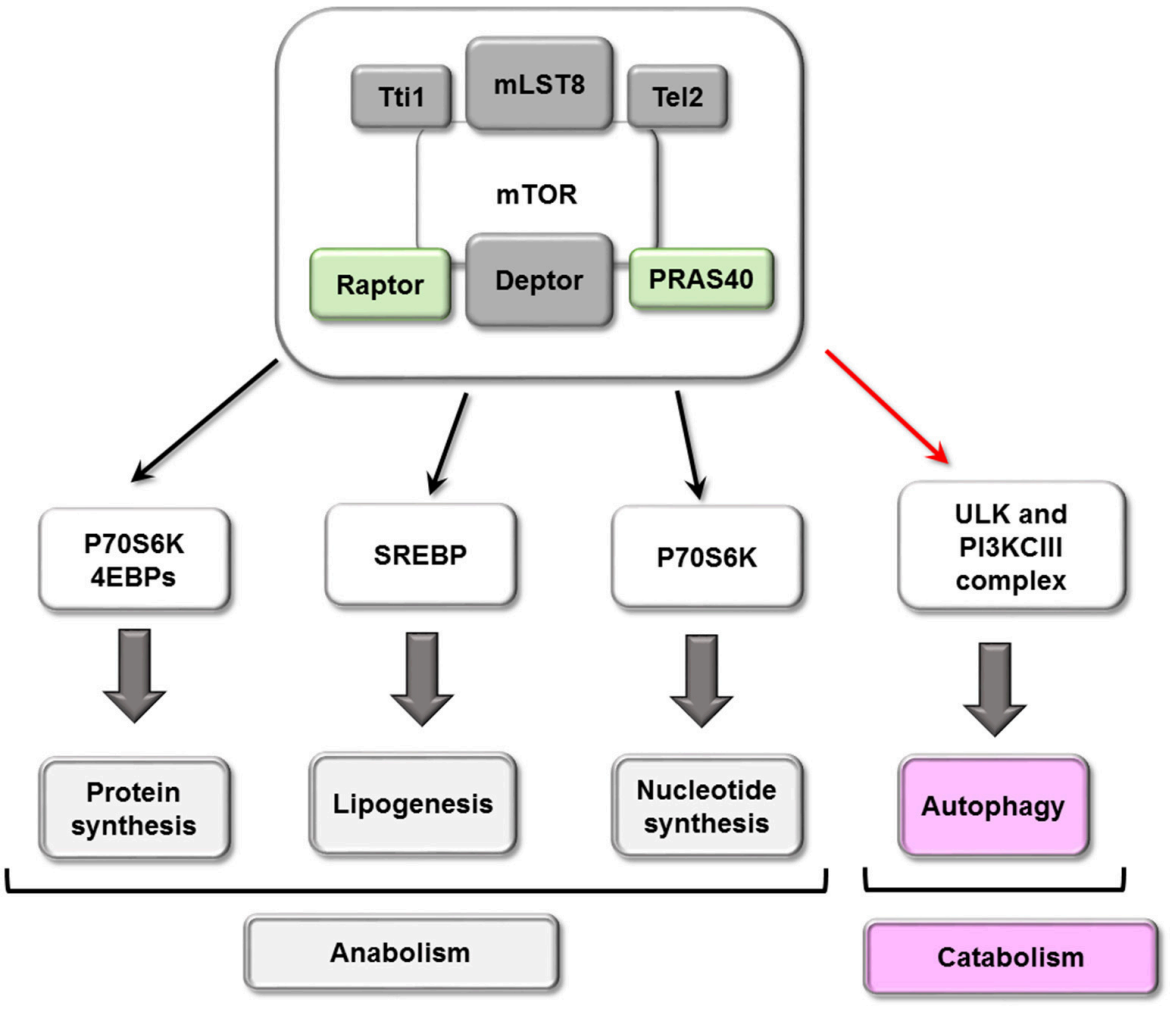
Cellular processes mTORC1 mediated. mTORC1 play a central role in cellular metabolism controlling some anabolic (synthesis of proteins and nucleotide, lipogenesis) and catabolic (autophagy) processes.

mTORC1 inhibits autophagy through several mechanisms, the first of which involves its phosphorylation of ULK1/2 and ATG13, dissociating and inactivating the pro-autophagy complex ULK [composed of ULK1/2, ATG13, ATG101 and RB1-inducible coiled-coil protein 1 (RB1CC1)/FIP200]. Secondly, mTORC1 can phosphorylate ATG14 and UVRAG, which inactivates phosphatidylinositol 3-kinase class III (PI3KCIII) complex during initial (composed of PIK3C3/Vps34, Beclin-1, PIK3R4/Vps15, ATG14 and AMBRA1) and maturing stages (composed of PIK3C3/Vps34, Beclin-1, PIK3R4/Vps15 and UVRAG) of autophagosomes (Nakamura and Yoshimori, 2017). Conversely, both ULK and PI3KCIII complexes are activated by AMPK-dependent phosphorylation (Kim et al., 2011, 2013; see scheme in Figure 3). A third mechanism involves mTORC1 phosphorylation of transcription factor TFEB, which prevents its translocation to the nucleus and therefore the transcription of several ATGs and lysosomal proteins (see scheme in Figure 4).

## Role of mTOR in ischemia/hypoxia

### Different approaches *in vitro* and *in vivo*

Cerebral ischemia, also known as ischemic stroke, is a sudden vascular accident that occurs when blood flow is reduced (transiently or permanently) to any cerebral artery by a clot, thrombus or atherosclerotic plaque. Globally, it is the second leading cause of death and the leading cause of adult long-term disability, and thus has a great social and economic impact (Thrift et al., 2014). Age is one of the most influencing factors related to incidence of stroke (Benjamin et al., 2017), and due to longer life expectancy, the incidence of ischemic stroke will increase over time. There does not currently exist any pharmacological treatment to reduce brain damage after stroke; the only therapeutic approaches are reperfusion by thrombolytic administration (only rtPA is approved) or surgery. However, not all stroke patients are appropriate for reperfusion, and only 10-20% of stroke patients receive rtPA (Lees et al., 2010), mainly because the window to restore blood flow to a cerebral artery is short (about 3–4 h from first symptoms) and the risk of cerebral hemorrhage is high after this time (Lenglet et al., 2014). The scientific community is making an intense effort to identify possible therapeutic targets to reduce ischemic damage and improves patient quality of life and to potentiate the effects of reperfusion in suitable patients. However, to date the pharmacological approaches that have been shown to be neuroprotective, reducing damage after stroke in animal models, have failed in humans (Cheng et al., 2004).

There are two different approaches to analyze the effects of ischemia and test potential neuroprotective compounds. The most commonly used *in vitro* model depends on oxygen and glucose deprivation (OGD) of primary cultured neurons or glial cells. This allows for precise control of duration of OGD exposure, permitting the study (even at molecular levels) of ischemia in each neural cell type at different times of injury. The most common *in vivo* model used is occlusion of middle cerebral artery (MCAO), which can mimic patients that have been treated after stroke (transient MCAO; tMCAO), or those who were not treated (permanent MCAO; pMCAO).

After hypoxia or ischemic conditions, the brain suffers a series of events collectively called “ischemic cascade,” including energy failure, excitotoxicity, neuroinflammation and delayed neuronal death by apoptosis (Pérez-Alvarez and Wandosell, 2013; Perez-Alvarez and Wandosell, 2016). The reduction in blood flow to the brain decreases availability of nutrients, oxygen, and energy, compromising neural cell viability, and the degree of cell damage depends on the duration and severity of ischemia. Accordingly, the brain is especially sensitive to energy and nutrient fluctuations for several reasons: (1) the brain is highly dependent on glucose and oxygen from the blood (it is estimated that it consumes more than 50% of total glucose in the body); (2) neurons use glucose as a primary energy source (although astrocytes can partially supplement nutrient requirements for neurons); and (3) release of energy from glucose relies on oxidative metabolism. Thus, it is reasonable to conclude that mTORC1 may play a key role after ischemia, in the balance between anabolic and catabolic processes needed to protect neurons from death.

Some studies have confirmed that after brain ischemia, the reduction (via blockage) of oxygen, glucose and growth factors triggers a decrease in mTORC1 activity. A dramatic inhibition of neuronal mTORC1 has been described after ischemia, through diminished activity of the PI3K/Akt pathway in both *in vitro* and *in vivo* models (Dutta et al., 2015; Mateos et al., 2016). Consequently, evidence supports lower mTORC1 phosphorylation of p70S6K, with a subsequent inhibition of its activity (Hwang and Kim, 2011; Dutta et al., 2015; Mateos et al., 2016). Studies with p70S6K1/2^−/−^ mice confirmed a role for this protein in brain protection after pMCAO (Pastor et al., 2009).

**Figure 5:**
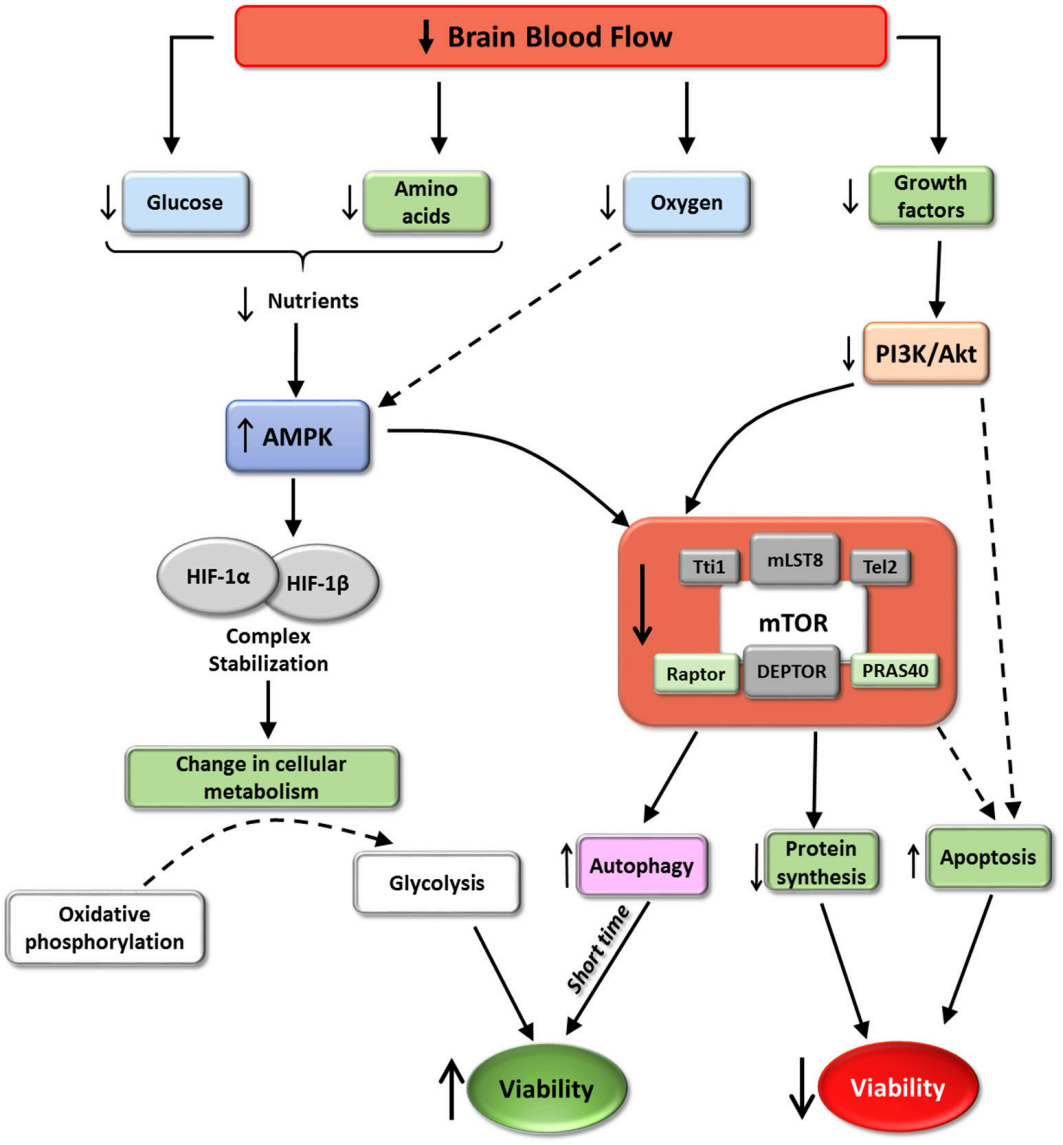
mTORC1 and cerebral ischemia. The scheme represent some of the effects of cerebral ischemia on mTORC1 activity according with the available data. Ischemia induces mTORC1 inhibition. These situation promotes a reduction in protein synthesis and an increase autophagy and in some situations apoptosis. The increment in autophagy, almost at short time, may induce an increment in cell viability. However, autophagy imbalance or ischemic persistence may trigger apoptosis. The balance of this process via mTORC1, determinates the final destiny of hypoxia affected cells.

Glial cells are also affected by cerebral ischemia; after OGD/reperfusion, activation of the mTOR pathway is key to restore proliferation, migration and production of inflammatory mediators by astrocytes and microglia (Chong et al., 2007; Li et al., 2015). In pathological situations, a decrease in mTORC1 activity using rapamycin reduced glia scar formation after traumatic spinal cord injury, preventing astrocytes growth and proliferation (Goldshmit et al., 2015). Anoxic conditions reduce p70S6K1 mRNAs in astrocyte cultures, leading to cell death after OGD by an unbalance of pro- and anti-apoptotic factors and increased ROS (Pastor et al., 2009). Therefore, some negative aspects of ischemic injury related to cellular death are triggered by deregulation of PI3K/Akt/mTORC, and increased activity of this pathway has proven to ameliorate the damage (see scheme in Figure 5).

It is well known that ischemic conditions induce transcription of HIF-1, a transcriptional factor that is a heterodimer composed of HIF-1α and HIF-1β. HIF translocates to the nucleus and can reprogram gene expression to facilitate cell survival in hypoxic conditions. The main process triggered by HIF consists of a shift from oxygen-consuming oxidative phosphorylation to oxygen-independent glycolysis, ensuring continued generation of ATP during hypoxia (reviewed in Chen and Sang, 2016). HIF-1 activity is determined by HIF-1α, thus the amounts of available HIF-1α are precisely regulated by a nutrient- and oxygen-dependent mechanism. In normal conditions, physiological oxygen levels induce HIF-1α ubiquitination and proteasomic degradation. In contrast, the low levels of oxygen, glucose and amino acids during hypoxia induce AMPK activity, stabilizing HIF-1α in combination with chaperones (such as Hsp90), increasing HIF-1 activity. A connection between HIF-1 and mTORC1 has been reported: Isoflurane preconditioning, which reduces neurological deficits, infarct volume, brain edema and apoptosis after ischemia induction, up-regulated HIF-1α expression via Akt/mTOR/p70S6K activation (Yan et al., 2016). Although translation of HIF-1α is not affected by inhibition of mTORC1, some reports have described an increment of HIF-1α synthesis when mTORC1 activity was increased (Hudson et al., 2002; Brugarolas et al., 2004; Bernardi et al., 2006; see scheme in Figure 5).

### Some neuroprotective agents regulate mTOR activity

Many data support the hypothesis that PI3K-Akt-mTORC1 represents a major contribution, in almost all cell types, to controlling cell survival and proteostasis. In fact, in many neurodegenerative diseases, dysfunction of this pathway has been reported (Heras-Sandoval et al., 2014; Perluigi et al., 2015; Zhao et al., 2015). As a consequence, recovery or enhancement of this pathway has been proposed as a therapeutic strategy, with targets ranging from PI3K, Akt, and GSK3 to mTORC1 (Shimobayashi and Hall, 2014).

Considering the diversity of pathologies affecting the CNS and the broad processes regulated by mTORC, below is presented an analysis of the role of mTORC1 as a regulator of macro-autophagy and neuroprotection, using an acute damage disease model, such as ischemic stroke. The role of mTORC1 as a neuroprotective target in ischemia models is still a controversial matter. Some contradictory data have been reported in relation to beneficial or detrimental effects of autophagy induction in the context of cerebral ischemia (Buckley et al., 2014; Chauhan et al., 2015; Luo et al., 2015; Zhang et al., 2017). Indeed, beneficial effects have been described after ischemic brain injury by reducing autophagy (Guo et al., 2014; Wang et al., 2016; Jiang et al., 2017; Zhang et al., 2017). Moreover, have been reports some improvement in pathophysiological parameters by increasing autophagy before ischemic damage (Yin et al., 2012; Chauhan et al., 2015; Luo et al., 2015). The use of rapamycin and chloroquine in mouse models to induce or block autophagy, respectively, immediately after stroke indicated that both increase and decrease of autophagy reduced the size of the infarct area (Buckley et al., 2014). However, only increased autophagy before damage or at the same time, improved neurological score, and reduced neuronal death to a greater extent (Buckley et al., 2014; Chauhan et al., 2015).

Some data using an “ischemic tolerance” or “ischemic preconditioning” paradigm in the heart indicate that sublethal ischemic insults prior to real damage may be protective, permitting the hypothesis that a protective endogenous mechanism triggered after a moderate brain ischemia may make tissue resistant to a more severe ischemic insult (Lee et al., 2000). Some evidence supports that the mTORC1 pathway is a mediator of this neuroprotective process by inducing autophagy, and Tsc1 has been revealed as a key player. Overexpression of Tsc1 conferred protection to hippocampal neurons after OGD by inducing efficient autophagic flux. However, downregulation of TSC1 triggered autophagosome accumulation, compromising autophagy (Papadakis et al., 2013). However, not only has the role of mTORC1 in autophagy been reported to be neuroprotective in the paradigm of ischemic tolerance, but its inhibition promoted this protective mechanism in part through changes in translation (Wouters and Koritzinsky, 2008).

Treatment with rapamycin after 1 h of hypoxic or ischemic conditions in an *in vitro* model of OGD improved neuronal survival after damage (Fletcher et al., 2013), and rapamycin preconditioning before tMCAO improved neurological deficits and reduced infarct area and brain edema (Yin et al., 2012). Reports on the effects of pre-ischemic rapamycin administration have revealed a parallel between increased autophagy and decreased mTORC1 activity, diminishing apoptosis, significantly reducing infarct area and oxidative stress, and improving behavioral outcomes after ischemia (Chauhan et al., 2011; Chong et al., 2013).

Nevertheless, as we have commented previously, some reports demonstrated detrimental effects of autophagy induction in rodents models of cerebral ischemia (Guo et al., 2014; Jiang et al., 2017; Zhang et al., 2017). These controversial results must be analyzed carefully taking into account the moment in which autophagy is induced or blocked in animal models. Autophagy induction before or just after ischemic damage, result in beneficial effects measured as improvement of neurological score, reduction of infarct area or diminution of brain edema (Yin et al., 2012; Chauhan et al., 2015; Luo et al., 2015). Opposite results have been obtained when autophagy induction was later (after almost 1 h of ischemic damage) (Guo et al., 2014; Jiang et al., 2017; Zhang et al., 2017).

In some mouse models, treatment with agents that increase the activity of the PI3K/Akt/mTORC pathway (before/after induction of ischemia) has been shown to be neuroprotective, reducing ischemic damage and improving neurological score (Guo et al., 2014; Zheng et al., 2014; Lisi et al., 2015; Mateos et al., 2016; Chen et al., 2017).

Taking into account these data, we can conclude that after ischemic damage the accessibility of growth factors decrease, the canonical pathway PI3K/Akt/mTORC1 is downregulated and consequently autophagy may be enhanced.

However, authophagy induction before ischemia makes neural tissue more resistant to damage, while a sustained increment of autophagy or a greater inhibition of mTORC1 or lysosomal proteases activity after ischemia induce detrimental effects related with neuronal and/or glia, survival.

In conclusion autophagy could be considered an endogenous protective mechanism, at some extent, triggered after ischemic damage, because downregulation of PI3K/Akt/mTORC1 pathway. However, sustained increment of autophagy over the time or an excess of “levels” of autophagy, or sustained inhibition of PI3K/Akt/mTORC1 are detrimental for neuronal survival. This highlights the importance of a fine tune control of both: the autophagy process and mTORC1 activity, to maintain neuronal viability in ischemic conditions.

An important neuroprotective element used in ischemia models is estradiol, which has been reputed to reduce ischemic damage either pre- or post-insult. In general, estrogens function via different types of response: “genomic actions” regulated by classical estrogen receptors (ERs); “non-genomic” or “rapid actions” initiated by ERs and membrane-associated receptors; and ER-independent mechanisms, such as the antioxidant effects reported by estradiol (for a review see: Mann et al., 2007; Arevalo et al., 2015). In addition to the “classical” nuclear actions, rapid estrogen effects have been described that occur within minutes and thus cannot be attributed to genomic mechanisms (Toran-Allerand et al., 2002; Raz et al., 2008). In fact, estradiol may activate MAPK/Erk (Singh et al., 1999), PI3K (Honda et al., 2000) and Akt (Cardona-Gomez et al., 2004; Mendez et al., 2006), among other cytoplasmic elements, using either classical or membrane-associated receptors (for review see: Arevalo et al., 2015). It has been reported that estradiol may have therapeutic effects in ischemia models, as injection (even post-ischemia) reduced the damage area, at least in part due to activation of the PI3K/Akt pathway (Koh, 2007; Pérez-Álvarez et al., 2012; Pérez-Alvarez and Wandosell, 2013). This strongly support the hypothesis that estradiol may control the activity of mTORC1 via cytoplasmic signaling (Varea et al., 2013).

Another important neuroprotective mechanism after cerebral ischemia is angiogenesis, the generation of new capillaries, which has been revealed to be a critical contributor to tissue recovery. Postmortem analysis of ischemic brain tissue revealed a significant increase in the number and size of microvessels in affected areas compared to the contralateral hemisphere, associated with longer patient survival (Krupinski et al., 1994). Angiogenesis after ischemic insult contributes to improved cerebral blood flow in the penumbra and reduces brain damage. mTORC1 has been identified as a potent mediator in this process, as mTORC1 activation increases VEGF levels (via the PI3K/Akt/mTORC1 pathway) by inducing its transcription in neurons (Chen et al., 2012; Mateos et al., 2016). This angiogenic enhancement reduces ischemic damage by decreasing the affected area and resulted in a higher neurological score in animal models of stroke (Mateos et al., 2016). Indeed, it has been reported that rapamycin administration reduced VEGF induction, preventing angiogenesis and increasing apoptotic cell death (Stahl et al., 2008).

At a pharmacological level, the role of statins must be mentioned, having been reported to have pleiotropic effects on neuroprotection. Statins inhibit HMG-CoA reductase, generating not only a reduction of cholesterol but also other intermediate metabolites essential for the formation of isoprenoids, such as geranyl pyrophosphate or farnesyl pyrophosphate, that are incorporated into such proteins as the Ras and RhoA families, among others, which play key roles in cellular signaling.

A second described effect for statins is enhancement of angiogenesis. Atorvastatin promoted angiogenesis, boosting functional recovery after stroke through a mechanism increasing VEGF, BDNF, eNOs, and some synaptic proteins such as glutamate receptor GluN1 or GluN2B (Cespedes-Rubio et al., 2010; Gutierrez-Vargas et al., 2014) in a mouse MCAO model (Brouet et al., 2001; Chen et al., 2003). Some of these actions are mediated by activation involving, directly or indirectly, the PI3K/Akt/mTORC1 pathway (Kureishi et al., 2000; Yang et al., 2015).

## Conclusions

PI3K/Akt/mTORC1 is an important pathway that is implicated in some neurodegenerative diseases and also in recovery from ischemic stroke. Thus, genetic or pharmacologic enhancement of this pathway should be considered a potent neuroprotective tool, though the exact role of mTORC1 after stroke and its connection with regulation autophagy must be more carefully investigated to clarify controversial data. For instance, temporal studies are needed since it is very possible that the role of mTORC1 and autophagy depends on duration as well as the severity of nutrient/growth factor/oxygen deprivation. Accordingly, more work should be carried out to clarify whether alternative methods to enhance neuronal autophagy have additional therapeutic benefits after brain ischemia.

## Author contributions

MP-A and FW, “The mTORC: role in adult CNS”; MP-A and MV, “Signaling pathways downstream of mTORC”; MP-A and IB-C, “Signaling pathways downstream of mTORC”; MP-A, “Role of mTOR in ischemia/Hypoxia”; FW and MP-A, “Some neuroprotective agents regulates mTOR Activity”; FW, “Conclusion.” The figures has been designed and made by MV, FW, and MP-A.

### Conflict of interest statement

The authors declare that the research was conducted in the absence of any commercial or financial relationships that could be construed as a potential conflict of interest.
